# Divergent Climate Responses Decouple Stem Growth from Canopy Activity Across Mongolian Pine Plantations in Northern China

**DOI:** 10.3390/plants15213403

**Published:** 2026-11-02

**Authors:** Mingyong Li, Leilei Yang, Shenhao Song, Guangyou Hao

**Affiliations:** 1 CAS Key Laboratory of Forest Ecology and Silviculture, Institute of Applied Ecology, Chinese Academy of Sciences, Shenyang 110016, China; 2 Zhejiang Business Technology Institute, Ningbo 315012, China; 3 University of Chinese Academy of Sciences, Beijing 100049, China

**Keywords:** climate response, drought, stem growth, canopy activity, Mongolian pine plantations

## Abstract

Stem growth and canopy activity are two of the key processes in trees and are associated with one another. These two processes and their relationships may be altered under climate change, yet the underlying mechanisms by which climatic factors impact them remain unclear. This study examined stem growth and canopy activity and their responses to climate using tree-ring width data from 614 increment cores and satellite-based normalized difference vegetation index (NDVI) data from 17 Mongolian pine (*Pinus sylvestris* var. *mongolica* Litv.) plantation sites in northern China. Stem growth was not significantly (*p* > 0.05) correlated with canopy activity at both broad spatial and individual-site scales. Broadly, stem growth was primarily driven by temperature-induced water stress whereas canopy activity was constrained by growing season precipitation, causing a difference in the spatial patterns of these two processes across the Mongolian pine plantations. Locally, stem growth was more vulnerable to high-temperature-exacerbated drought stress during the growing season and more affected by pre-growing-season hydroclimatic variability than canopy activity, especially at more arid sites, which could decouple interannual correlations between the two processes. Our study reveals climate-driven decoupling between stem growth and canopy activity, complicating the estimations of overall carbon dynamics, and thereby underscores the necessity of comprehensively assessing forest dynamics and carbon sequestration.

## 1. Introduction

Climate change over recent decades has exerted significant impacts on the structure and function of forest ecosystems, resulting in biodiversity loss, productivity decline and other adverse effects [1,2,3,4,5]. The relationships between forest ecosystems and climate have been investigated extensively from the perspectives of both time and space [6]. Studies on the relationships between mean tree growth and mean climate conditions at broad spatial scales have found that higher growth occurs at wetter and warmer sites and lower growth at drier and colder ones, reflecting the predominant control of site-specific long-term climatic conditions on growth [7,8]. On the other hand, numerous studies have revealed interannual correlation patterns between tree growth and climate-related metrics during certain periods in many regions, underscoring the sensitivity of growth dynamics to short-term climate fluctuations, e.g., [9,10,11]. Furthermore, several studies assessed growth resilience to short-term extreme climate events, such as severe droughts, by calculating drought resistance and recovery indices, which reflect trees’ capacity to withstand extreme climatic conditions [12,13]. Therefore, examining tree growth–climate relationships that integrate both temporal and spatial aspects is critical for comprehensively revealing the impacts of climate on growth dynamics, but studies combining both perspectives with thorough investigations remain relatively few [7,14].

Growth–climate relationships, particularly those of stem growth and canopy activity with relation to climate, can be quantified by both dendroclimatological and remote sensing approaches [15,16]. Tree-ring analysis has been widely used to reveal long-term stem growth dynamics and their responses to climate change across various biomes [17,18,19,20,21,22,23,24,25,26]. In parallel, remotely sensed vegetation indices, commonly typified by normalized difference vegetation index (NDVI), can effectively monitor vegetation canopy activity at large spatial scales, and satellite-based NDVI has been widely used to examine canopy dynamics and their relationships with precipitation and temperature across various forest systems [16,27,28,29,30,31,32,33,34,35]. Since tree-ring parameters and NDVI reflect the growth responses of different organs (stems vs. canopies) to climatic factors, combining these two types of variables can provide complementary insights into tree growth dynamics.

Tree-ring parameters and remotely sensed vegetation indices are assumed to be associated with each other, since these two types of growth-related variables are sensitive to climate [16]. Several studies with regional to global scales have reported that there is a generally positive relationship between tree-ring growth and remote-sensing-related parameters (e.g., NDVI) during the growing season, reflecting changes in the same direction of these variables in response to changes in environmental conditions [36,37]. In contrast, recent research also found no obvious and even negative relationships between the two types of growth-related metrics [38]. The uncertainties that exist in relationships between tree-ring and remote sensing variables may be due to the fact that they, respectively, capture the dynamics of tree stems and canopies (mainly the foliage), which are regulated by different processes [16]. Carbon uptake by foliage (regarded as carbon sources) at the tree canopy obviously precedes carbon accumulated as wood (carbon sinks) at stems, which can weaken the coupling of canopy activities and tree-ring growth. Such a decoupling trend could be further aggravated, since a large proportion of carbohydrates obtained by foliage are used for respiration and other metabolic activities [39]. In addition, when a tree encounters environmental stress, it may allocate more resources to foliage restoration at the expense of fewer allocations to the trunk, since photosynthesis supported by foliage function is key for maintaining metabolism [40]. This trade-off in resource allocation between stems and leaves may also result in decoupling between tree-ring growth and the remote sensing vegetation index. Furthermore, non-preferential resource allocation for stem growth under more stressful conditions could lead to greater sensitivity of the tree-ring parameters to environmental factors relative to remote sensing indicators, which may complicate the relationships between these two types of variables and their associations with climate [16].

Diverse relationships between tree-ring-based parameters and remotely sensed vegetation indices across various forest ecosystems were thus attributed to distinct driving processes underlying stem growth and canopy activity [41,42]. This makes combinations of these two types of growth-related variables an effective approach to comprehensively revealing the growth dynamics of forests and their responses to environmental conditions, but related integrative studies are comparatively few [16]. Such research is critically needed for water-deficit forest ecosystems where severe water stress could intensify trade-offs in resource allocation between tree canopies and stems. This, in turn, may increase the uncertainty in the relationships between stem growth and canopy activity, as well as in the responses of the two processes to climate stressors, thereby complicating forest dynamics under climate change.

Mongolian pine (*Pinus sylvestris* var. *mongolica* Litv.) trees grow naturally in the Honghuaerji town of Hulunbuir in northeastern China and in its adjacent regions, and in recent decades they have been extensively planted to the south of their natural range to create shelter forests across northern China. Because of their drought tolerance and fast growth, Mongolian pine plantations play an important role in preventing desertification and elevating carbon storage [43]. However, these plantations have exhibited tree dieback and growth decline due to water deficit conditions in the region [43,44,45]. The deterioration of growth conditions has been further exacerbated by recent climate warming and drying, weakening the protective functions of the pine plantations and threatening their long-term sustainability. This highlights the urgent need to assess how plantation growth responds to climate change in such water-limited environments. Although Mongolian pine growth dynamics and underlying mechanisms have been examined at several sites in the region [46,47], growth characteristics at large spatial scales and their relationships with climate remain unclear. More importantly, most studies lack a comprehensive evaluation of growth performance across diverse organs (i.e., stems and leaves) [43,44,46]. Such single-organ approaches impede our understanding of how trees regulate aboveground carbon uptake and allocation under climate change. Given the extensive afforestation in northern China’s desertification prevention belt and the projected climate drying, clarifying the relationships between stem growth and canopy activity is essential for comprehensively revealing the dynamics, stability, and carbon sequestration capacity of plantations in water-deficit areas worldwide.

By combining tree-ring analysis and remote sensing, we investigated the dynamics of stem growth and canopy activity of Mongolian pine plantations and their responses to climate change in 17 sampling sites along a moisture gradient in northern China’s desertification prevention belt. We aim to reveal the associations between stem growth and canopy activity, as well as their dependence on climate drivers across different spatial scales in water-limited environments. Correspondingly, we put forward the following hypotheses: (1) the stem growth (represented by tree-ring width parameters) and canopy activity (growing season NDVI) of Mongolian pine plantations are positively correlated at both broad spatial and individual-site scales, given that they are both limited by hydroclimatic conditions in the study area; (2) the correlation patterns of stem growth with climate are consistent with those for canopy activity at both spatial scales; and (3) at the site scale, the pine plantations at drier sites are more sensitive to hydroclimatic variability than those at wetter sites.

## 2. Results

### 2.1. Variations in Stem Growth and Canopy Activity and Their Relationships Across Sites

**Figure 1:**
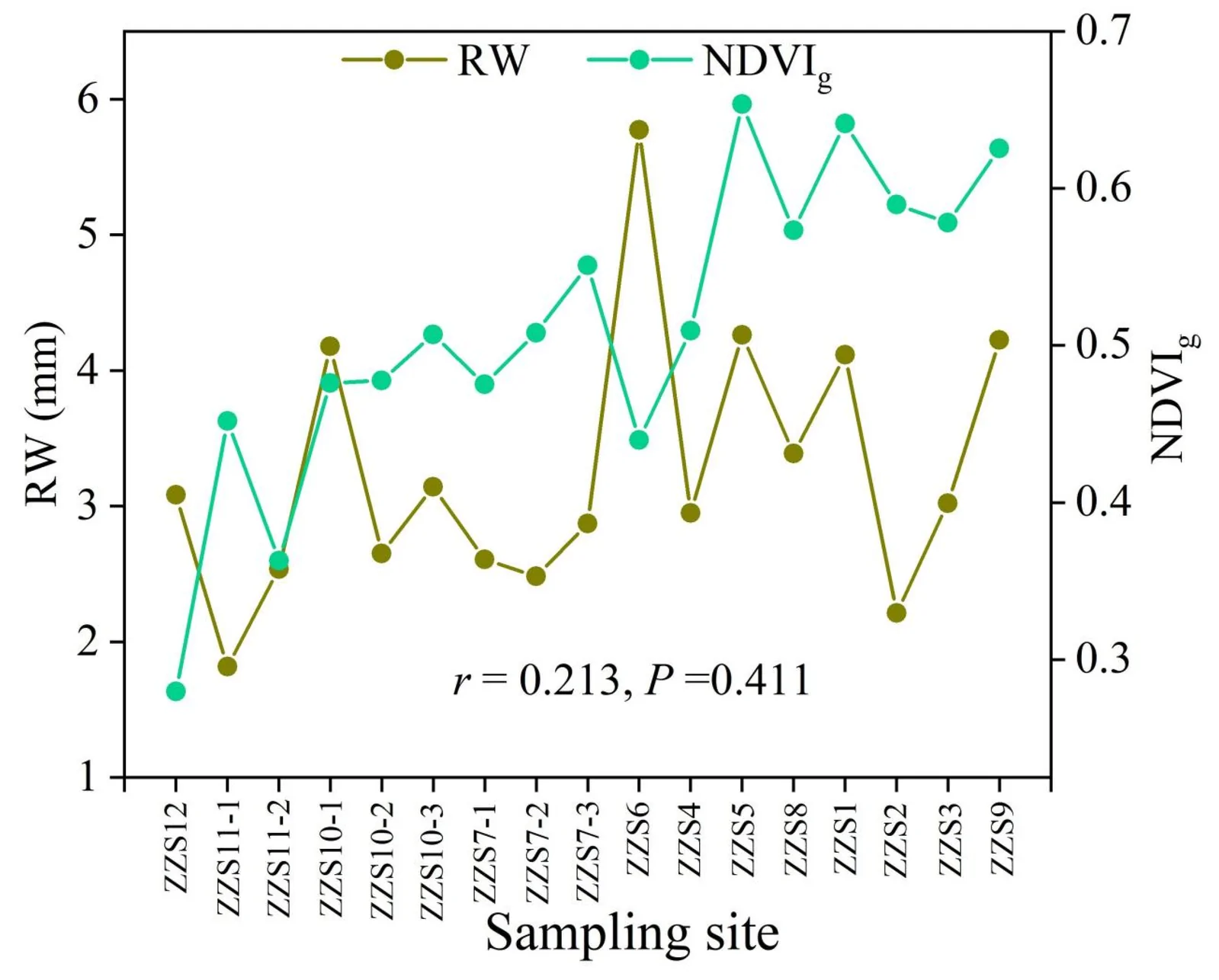
Changes in RW and multiyear mean growing-season (May–September) Normalized Difference Vegetation Index (NDVI_g_) along a precipitation gradient across the sampling sites of the Mongolian pine plantations. RW was used as an indicator of site-specific stem growth.

**Figure 2:**
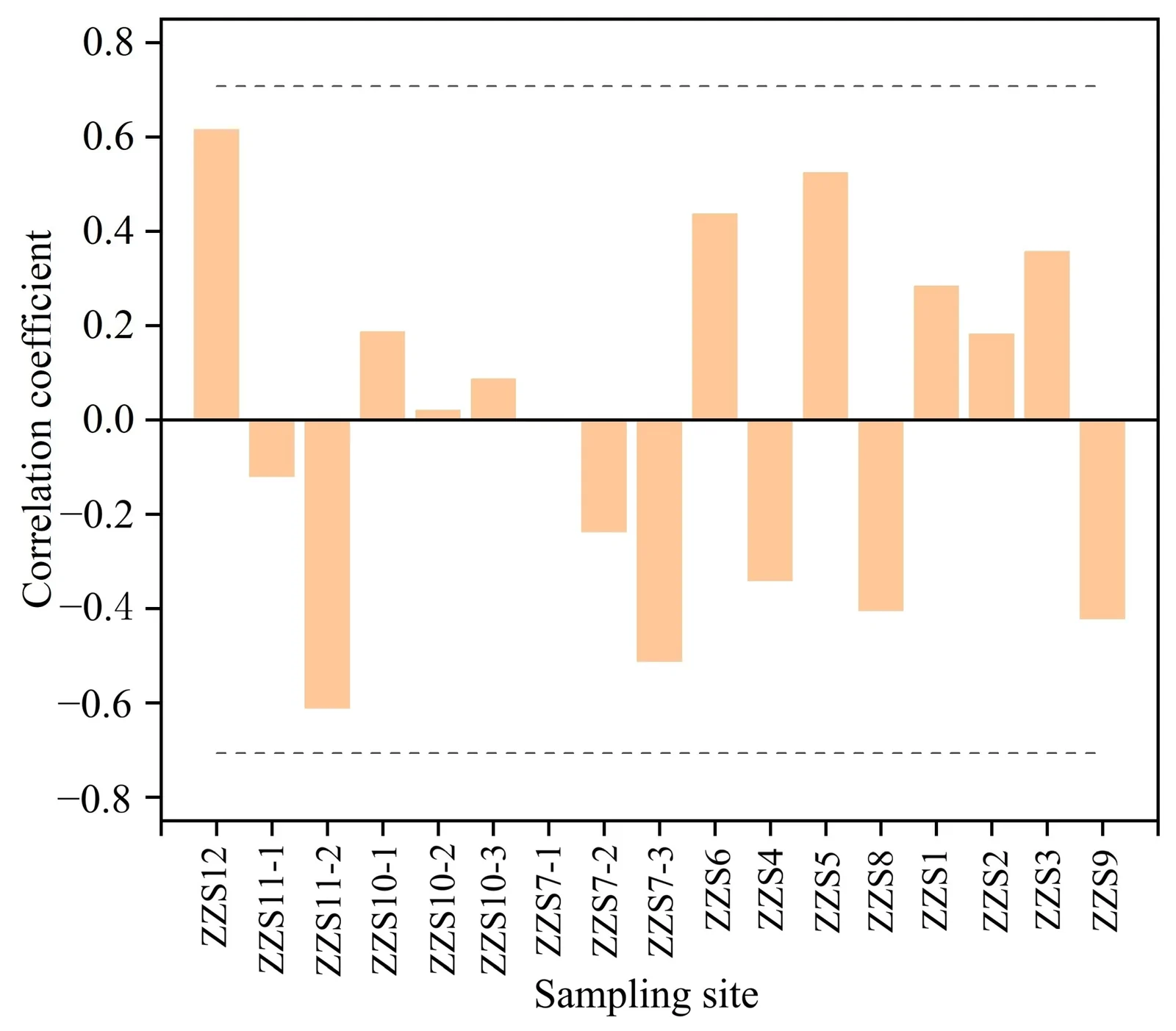
The correlations between the tree-ring width index (RWI) and growing-season (May–September) Normalized Difference Vegetation Index (NDVI_g_) at the 17 sampling sites of the Mongolian pine plantations. The horizontal dashed line indicates the correlation coefficient at the 0.05 significance level.

The stem growth of Mongolian pine plantations varied notably across the 17 sampling sites. The mean tree-ring widths during the periods with available data changed from 1.36 to 4.87 mm among the sites investigated (Table 1). The mean stem growths represented by averaged tree-ring widths of the first 8 years of tree-ring cores (RW), which reduced the potential age effects on growth behaviors, also varied between 1.82 and 5.77 mm (Figure 1). The variations in RW were highly consistent with those in mean basal area increment (BAI) over the periods with available tree-ring width data across the sites (*r* = 0.815; Figure S1a). In addition, the values of mean DBH ranged from 8.36 to 22.27 cm in these sites (Table 1). Consistent with variable stem growth, the canopy activity reflected by remotely sensed vegetation index also exhibited large variations among the sites studied. The multi-year average NDVI_g_ ranged from 0.280 to 0.654 across these sites (Figure 1). Correspondingly, a positive but insignificant correlation (*r* = 0.213; *p* = 0.411) was observed between RW and NDVI_g_ (Figure 1) at the broad spatial scale covering these sites. Such an insignificant relationship (*r* = 0.169; *p* = 0.517) was also shown between BAI and NDVI_g_ across the sites (Figure S1b). In addition, relatively weak relationships between the detrended tree-ring width index (RWI) and detrended NDVI_g_ were also observed at the individual-site scale, with the correlations between −0.612 and 0.615 (*p* > 0.05; Figure 2). The tree-ring width chronologies, which were widely used for analyzing growth–climate relationships, showed high reliability and substantial climate signals, with *R* ranging from 0.30 to 0.83 and EPS from 0.89 to 0.99 among the sites (Table 2). The MS of the chronologies across the sites ranged between 0.15 and 0.50.

### 2.2. Overall Responses of Stem Growth and Canopy Activity to Climate Across Sites

**Figure 3:**
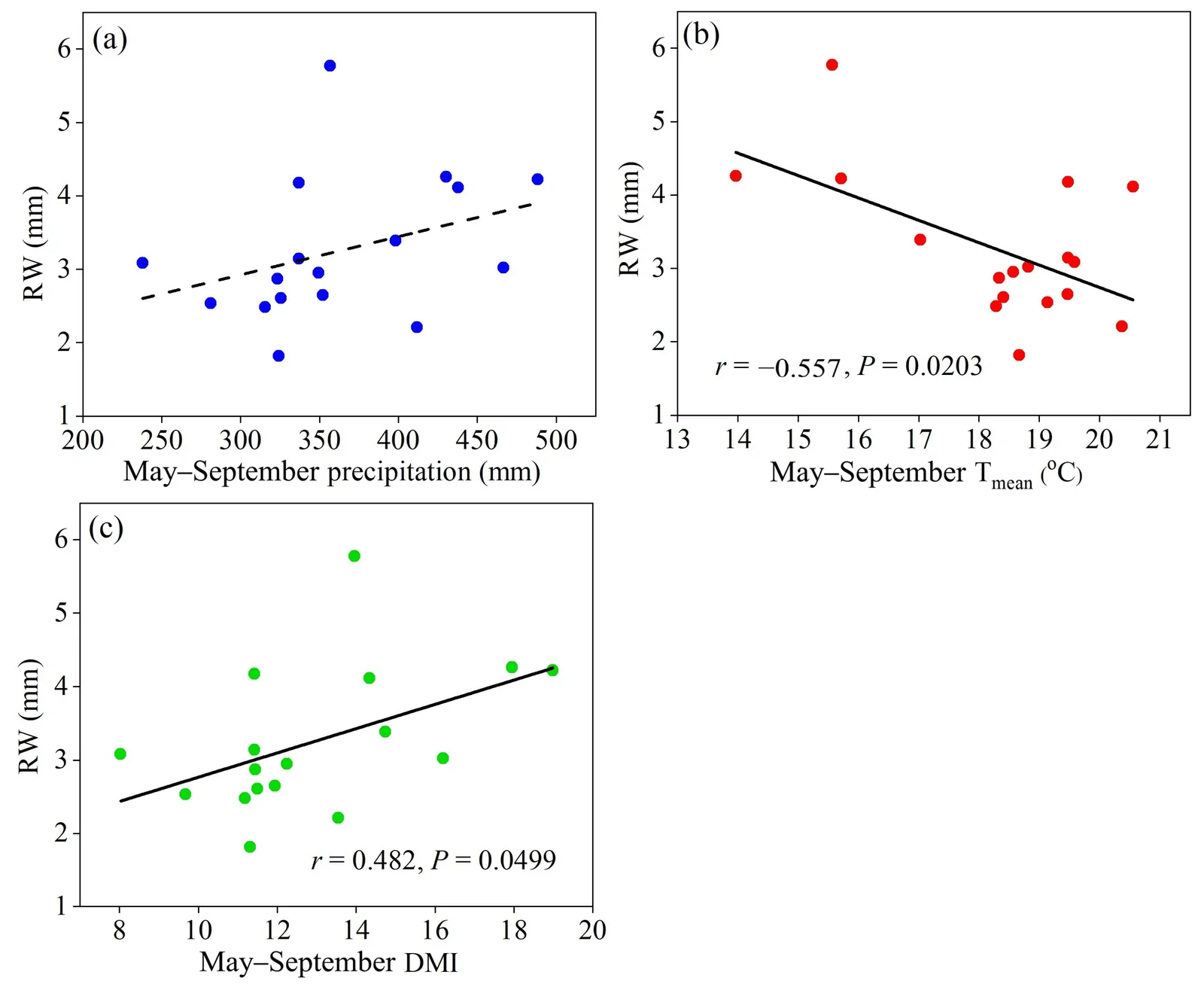
Scatter plots showing the relationships of RW with growing-season (May–September) (**a**) precipitation, (**b**) mean temperature (T_mean_), and (**c**) De Martonne drought index (DMI) across the sampling sites of the Mongolian pine plantations. RW was used as an indicator of site-specific stem growth. DMI was calculated as annual total precipitation (mm) divided by the sum of the mean annual temperature (°C) and 10 for the corresponding year. The linear trend is presented using a solid line when the *p* value is less than 0.05 and a dashed line when the *p* value is greater than 0.05.

**Figure 4:**
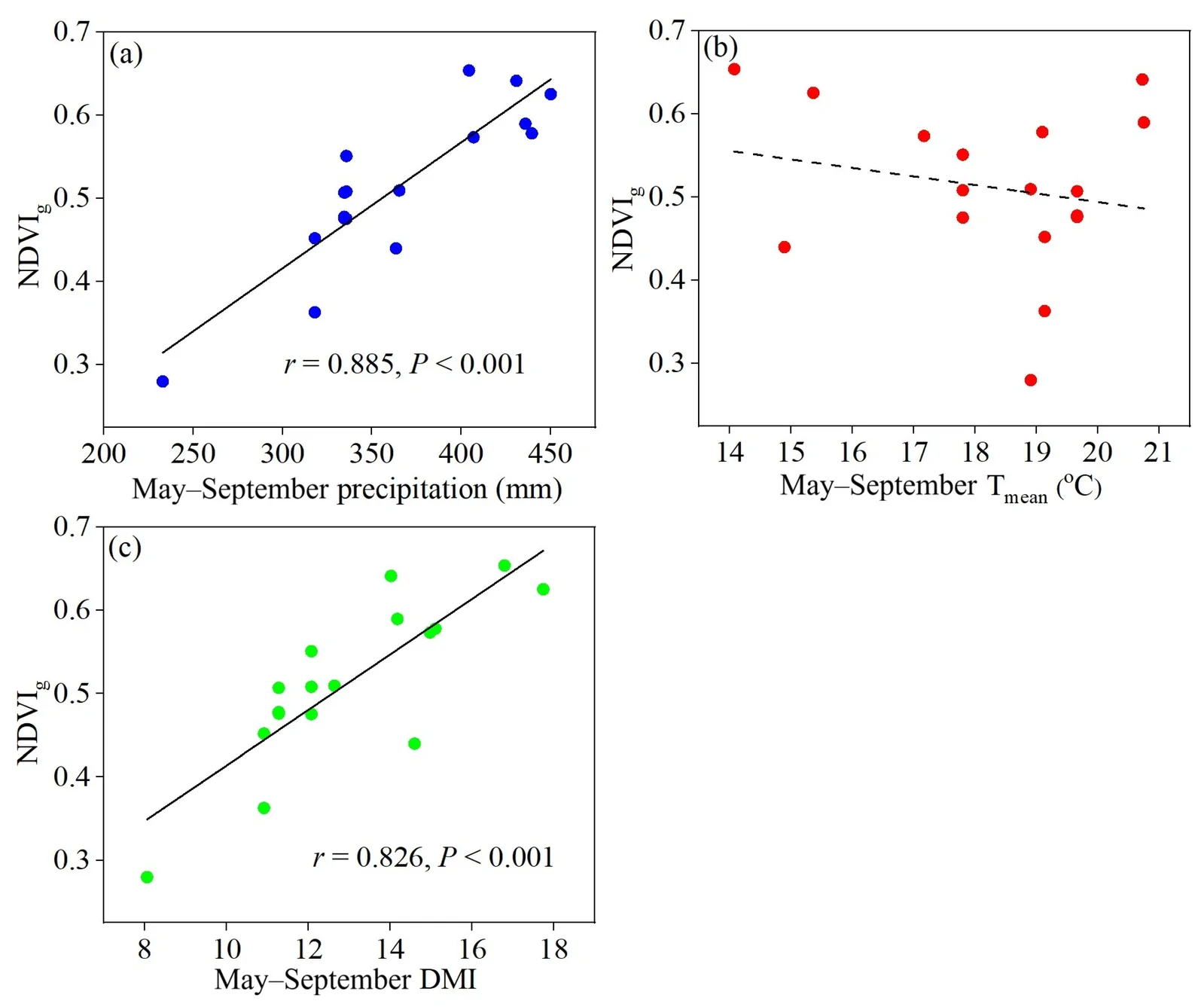
Scatter plots showing the relationships of growing-season (May–September) Normalized Difference Vegetation Index (NDVI_g_) with growing-season (**a**) precipitation, (**b**) mean temperature (T_mean_), and (**c**) De Martonne drought index (DMI) across the sampling sites of the Mongolian pine plantations. DMI was calculated as growing-season (May–September) total precipitation (mm) divided by the sum of growing-season mean temperature (°C) and 10. The linear trend is presented using a solid line when the *p* value is less than 0.05 and a dashed line when the *p* value is greater than 0.05.

The stem growth of Mongolian pine seemed to respond more strongly to temperature and drought than to precipitation across the broad region we studied. RW was not significantly correlated with growing season (May–September) precipitation across the sampling sites (Figure 3). However, a negative and significant (*p* < 0.05) correlation was observed between RW and growing season T_mean_. Furthermore, RW was found to be positively and significantly (*p* < 0.05) correlated with growing season DMI, a drought index incorporating both precipitation and temperature information. Inconsistently with RW, NDVI_g_ exhibited a strongly positive correlation with growing season precipitation across the sites investigated (*r* = 0.885; *p* < 0.001; Figure 4). Correspondingly, a strong correlation with growing season DMI was also observed for NDVI_g_ (*r* = 0.826). However, there was no obvious correlation between NDVI_g_ and T_mean_.

### 2.3. Site-Specific Responses of Stem Growth and Canopy Activity to Climate

**Figure 5:**
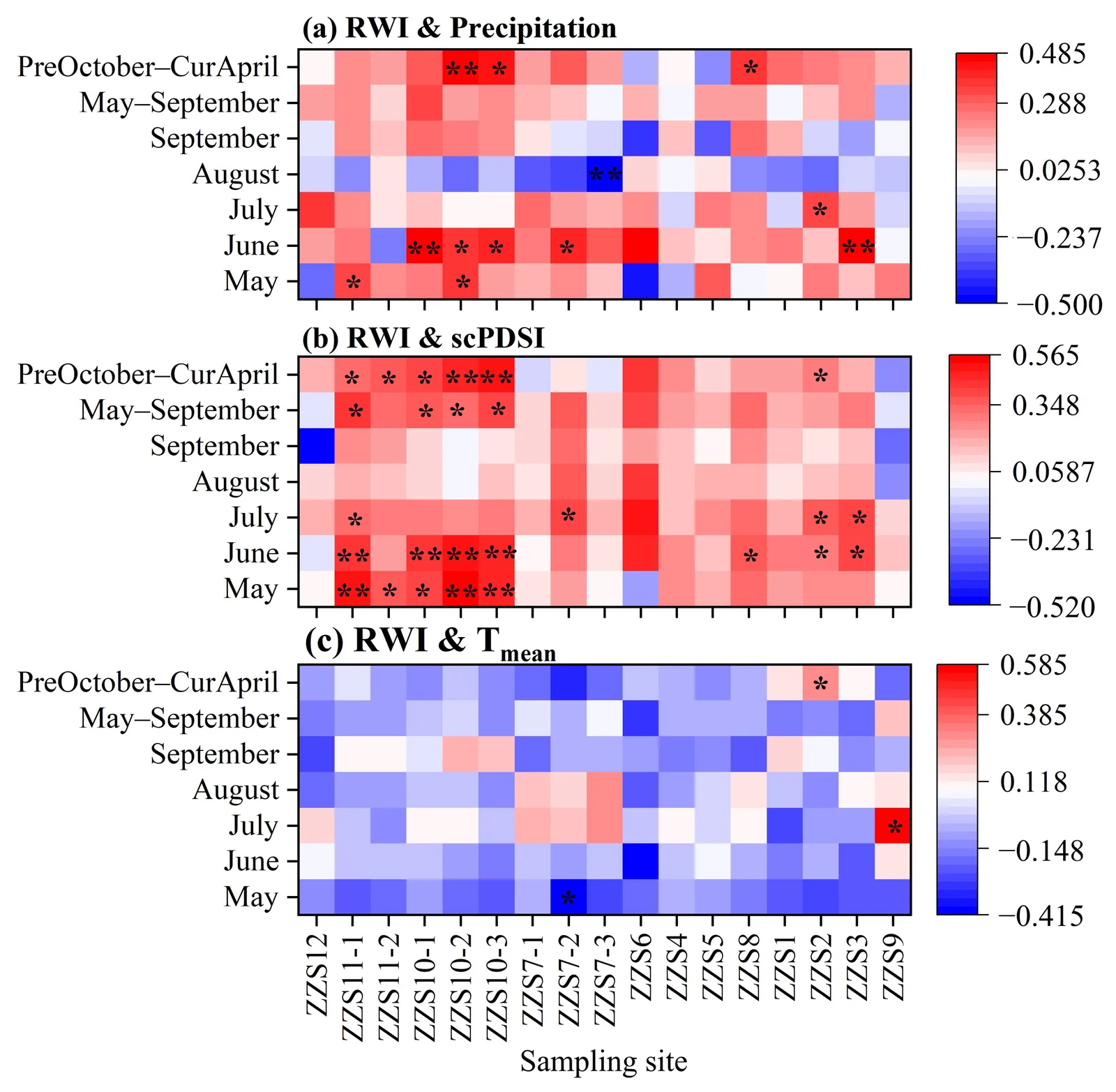
Correlations of the tree-ring width index (RWI) with monthly precipitation, self-calibrating Palmer drought severity index (scPDSI), and mean temperature (T_mean_) from May to September, as well as with growing-season (May–September) and pre-growing-season (PreOctober–CurApril) averages of these climate variables, during the periods with reliable RWI at each Mongolian pine plantation sampling site. * and ** denote that the correlations are significant at the 0.05 and 0.01 levels, respectively.

Consistent with the patterns observed across all sites, the impacts of hydroclimate on stem growth, amplified by temperature stress, also seemed to have also manifested in single sites. The RWI was generally positively correlated with growing season precipitation and scPDSI but negatively correlated with growing season T_mean_ in most of the sites (Figure 5). However, stronger positive correlations with RWI have been observed for scPDSI, which integrates information on both precipitation and temperature, than for precipitation alone. In addition, stronger impacts of water-related climate variables (precipitation and scPDSI) on RWI were observed in more arid sites (i.e., those with less precipitation), where significant (*p* < 0.05) correlations were more frequent compared to in more humid sites (Figure 5a,b). Meanwhile, significant (*p* < 0.05) positive relationships of RWI with climate variables mostly occurred in May and June. In addition to growing season climate, RWI also responded significantly (*p* < 0.05) to pre-growing-season precipitation (i.e., ZZS10-2 to ZZS10-3) and scPDSI (ZZS10-1 to ZZS10-3 and ZZS11-1 to ZZS11-2) at several dry sites (Figure 5a,b).

**Figure 6:**
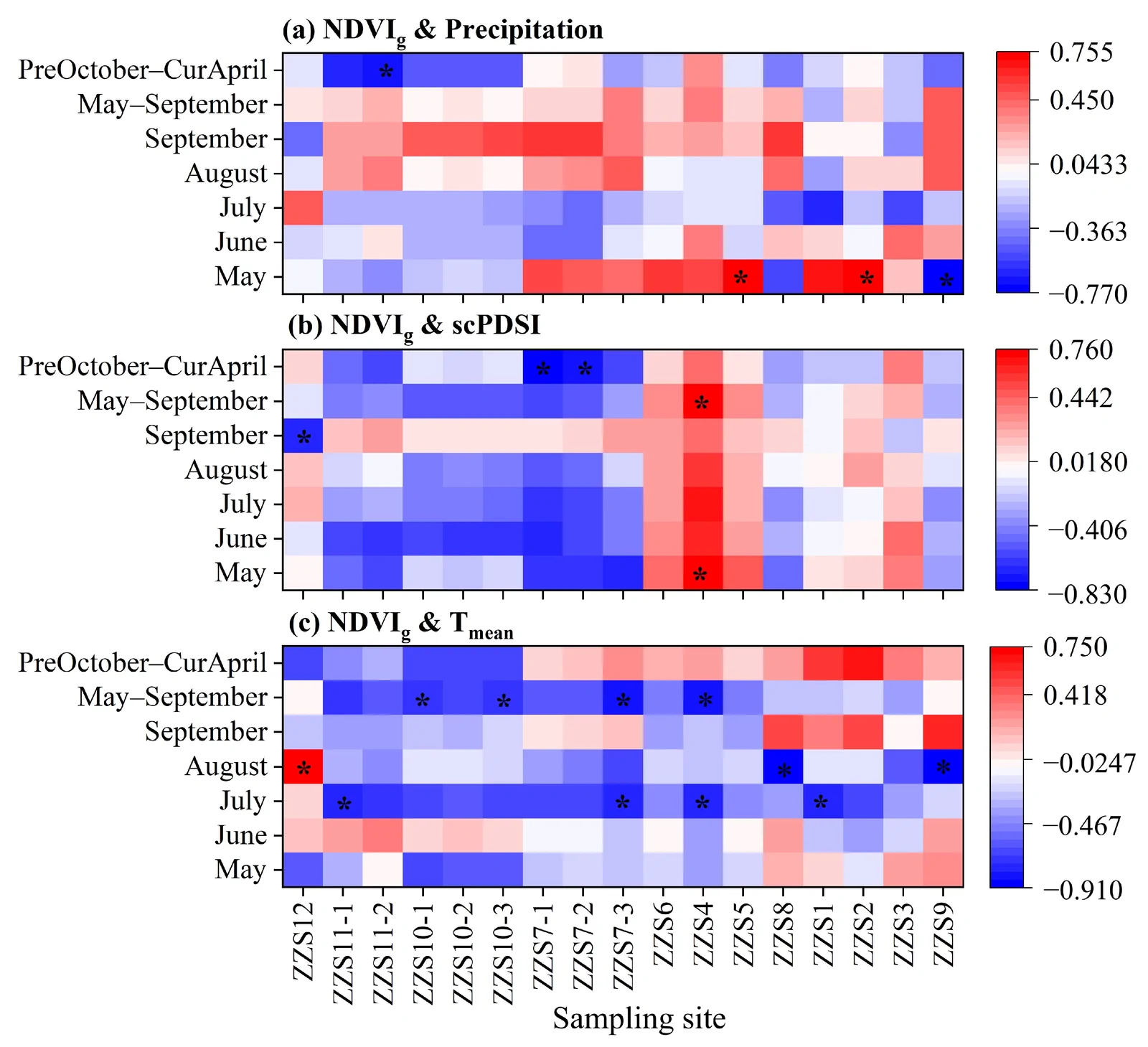
Correlations of the detrended Normalized Difference Vegetation Index during growing season (May–September; NDVI_g_) with monthly precipitation, self-calibrating Palmer drought severity index (scPDSI), and mean temperature (T_mean_) from May to September, as well as with growing-season (May–September) and pre-growing-season (PreOctober–CurApril) averages of these climate variables, during the period 2016–2023 at each Mongolian pine plantation sampling site. * denotes that correction is significant at the 0.05 level.

In contrast to the strong positive responses of canopy activity to hydroclimatic variables across all sites (Figure 4a,c), the positive relationships of canopy activity (reflected by NDVI_g_) with precipitation and scPDSI were weak in most individual sites, with significant (*p* < 0.05) correlations observed in only a few sites (Figure 6a,b). Furthermore, these positive correlations were also obviously weaker than those for RWI (Figure 5a,b and Figure 6a,b). Meanwhile, negative correlations between NDVI_g_ and both scPDSI and precipitation frequently occurred in some more arid sites. NDVI_g_ was generally negatively correlated with T_mean_ during growing season for the sampling sites investigated, with significant (*p* < 0.05) correlations observed in some sites (Figure 6c).

**Figure 7:**
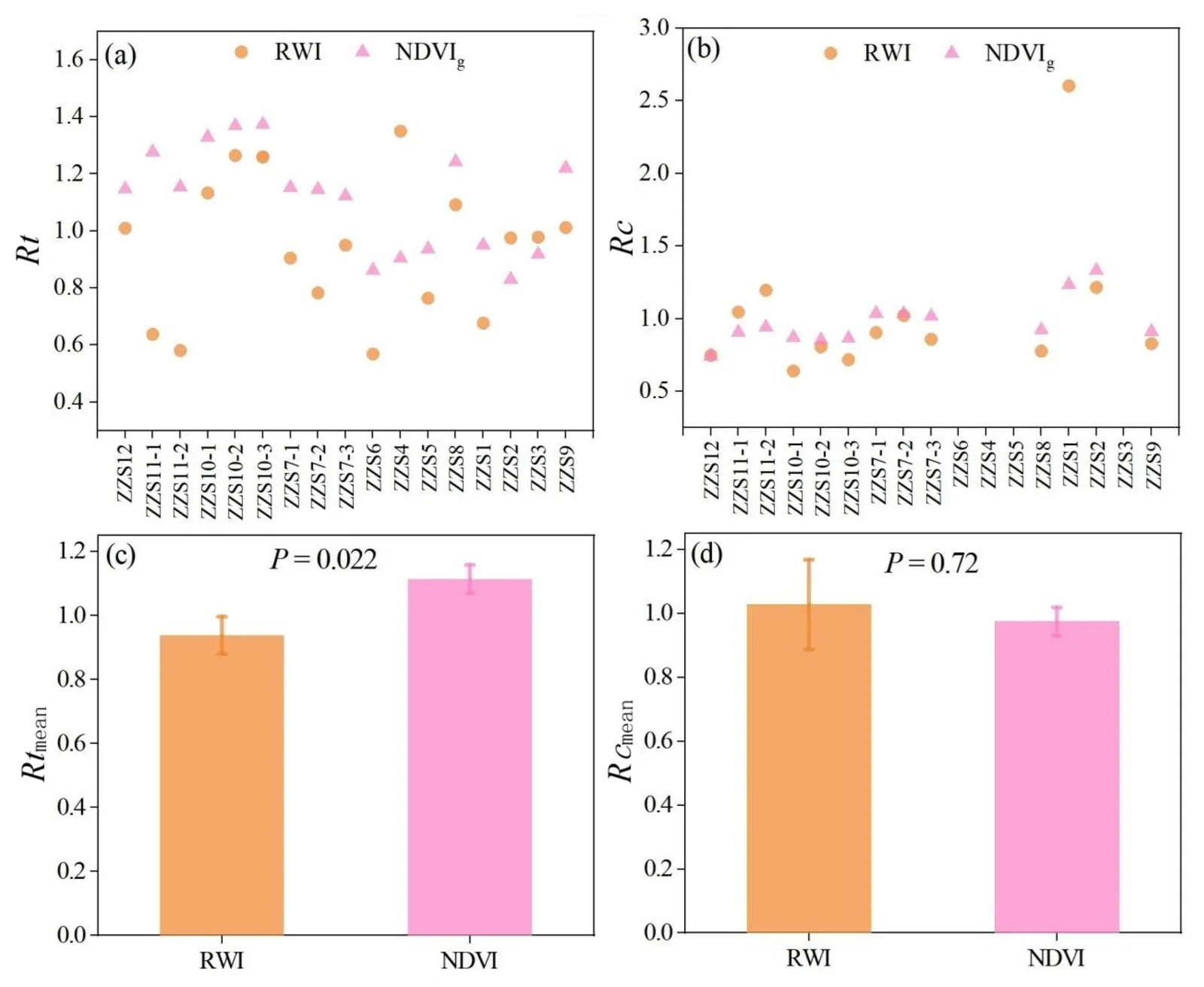
Drought resistance (*Rt*; (**a**)) and recovery (*Rc*; (**b**)) of the tree-ring width index (RWI) and the detrended growing-season (May–September) normalized difference vegetation index (NDVI_g_) at each of the sampling sites of the Mongolian pine plantations, and the mean resistance (*Rt*_mean_; (**c**)) and mean recovery (*Rc*_mean_; (**d**)) across all the sampling sites.

**Figure 8:**
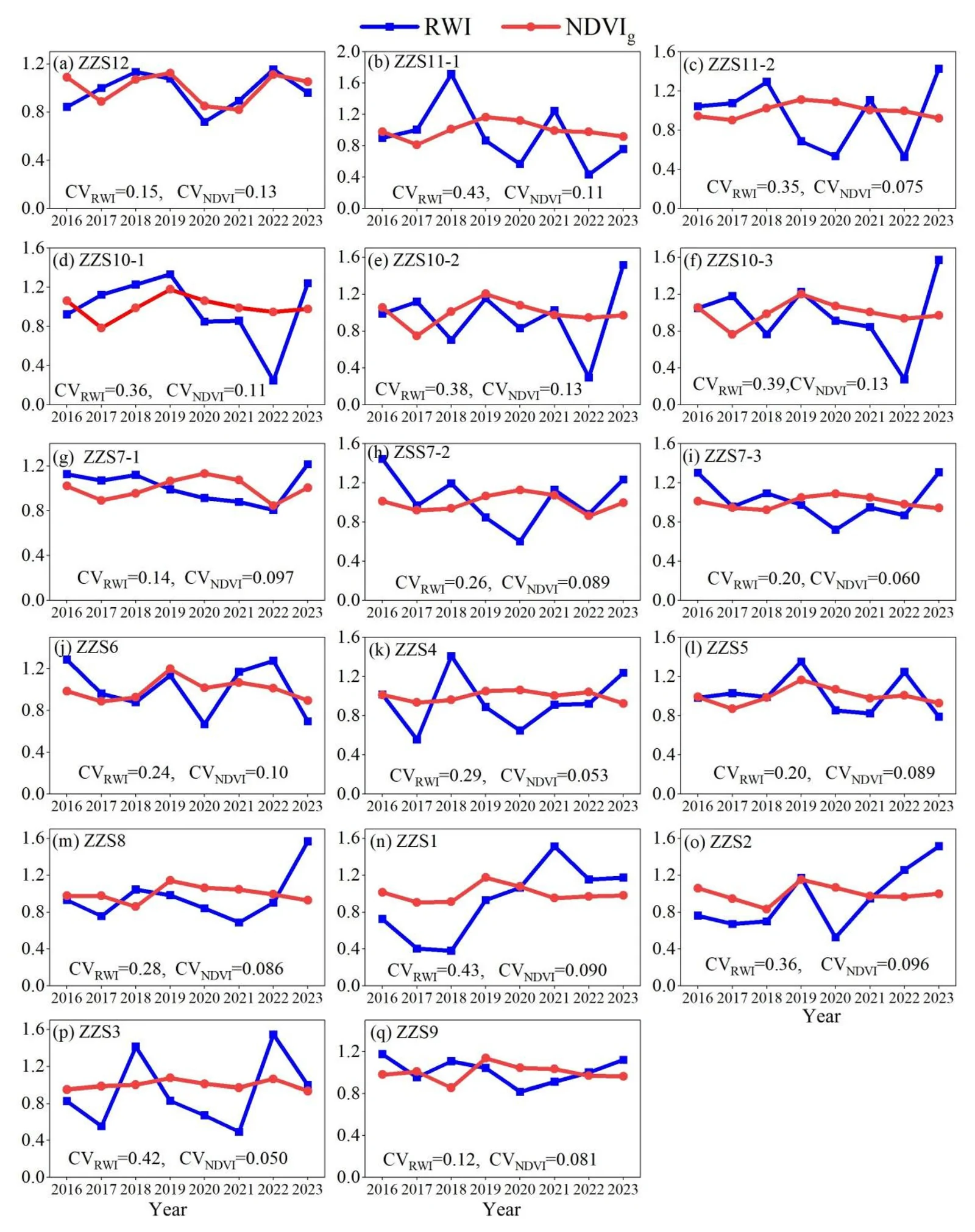
Comparisons of the tree-ring width index (RWI) and detrended growing-season (May–September) normalized difference vegetation index (NDVI_g_) over the common period 2016–2023 at each Mongolian pine plantation sampling site. CV_RWI_ and CV_NDVI_ denote the coefficient of variation of RWI and NDVI_g_, respectively.

Large differences were observed between stem growth and canopy activity in response to extreme droughts. The drought resistance (*Rt*) for RWI was lower than those for NDVI_g_ in most sites (14 of the 17 sites; Figure 7a). In addition, the mean drought resistance (*Rt*_mean_) of RWI across all sites was significantly (*p* < 0.05) lower than that of NDVI_g_ (Figure 7c). However, no obvious difference was found in mean drought recovery (*Rc*_mean_) across all sites between RWI and NDVI_g_ (Figure 7b,d). The coefficient of variation (CV) of RWI, which was closely associated with the impacts of extreme droughts, was greater than that of NDVI_g_ at all sites, with the largest difference occurring at the ZZS3 site (Figure 8). At this site, the CV of RWI was more than eight times that of NDVI_g_.

## 3. Discussion

Our study found no significant (*p* > 0.05) relationships between stem growth (reflected by tree-ring parameters) and canopy activity (represented by satellite-based NDVI during growing season) at both broad spatial and individual-site scales for Mongolian pine plantations in water-limited environments of northern China. This finding is not consistent with previous studies that reported strong positive correlations between tree-ring parameters and remotely sensed vegetation indices during growing season months at regional scales [16,36]. The weak association in dynamics between tree stems and canopies in our study area may be due to differences in their responses to climate. At the broad regional scale, stem growth appears to be more limited by high-temperature-induced water stress, whereas canopy activity is likely dominated by long-term precipitation deficit [48,49], suggesting a partial decoupling between the two processes. In addition, the greater sensitivity of stem growth than canopy activity to long-term climate variability and climate extremes at the individual-site scale may further amplify this divergence. In the following sections, we discuss the potential mechanisms underlying these divergent climate responses and their implications for the observed decoupling of stem growth and canopy activity in Mongolian pine plantations across northern China.

### 3.1. Divergent Climate Responses Decouple Stem and Canopy Dynamics Regionally

**Figure 9:**
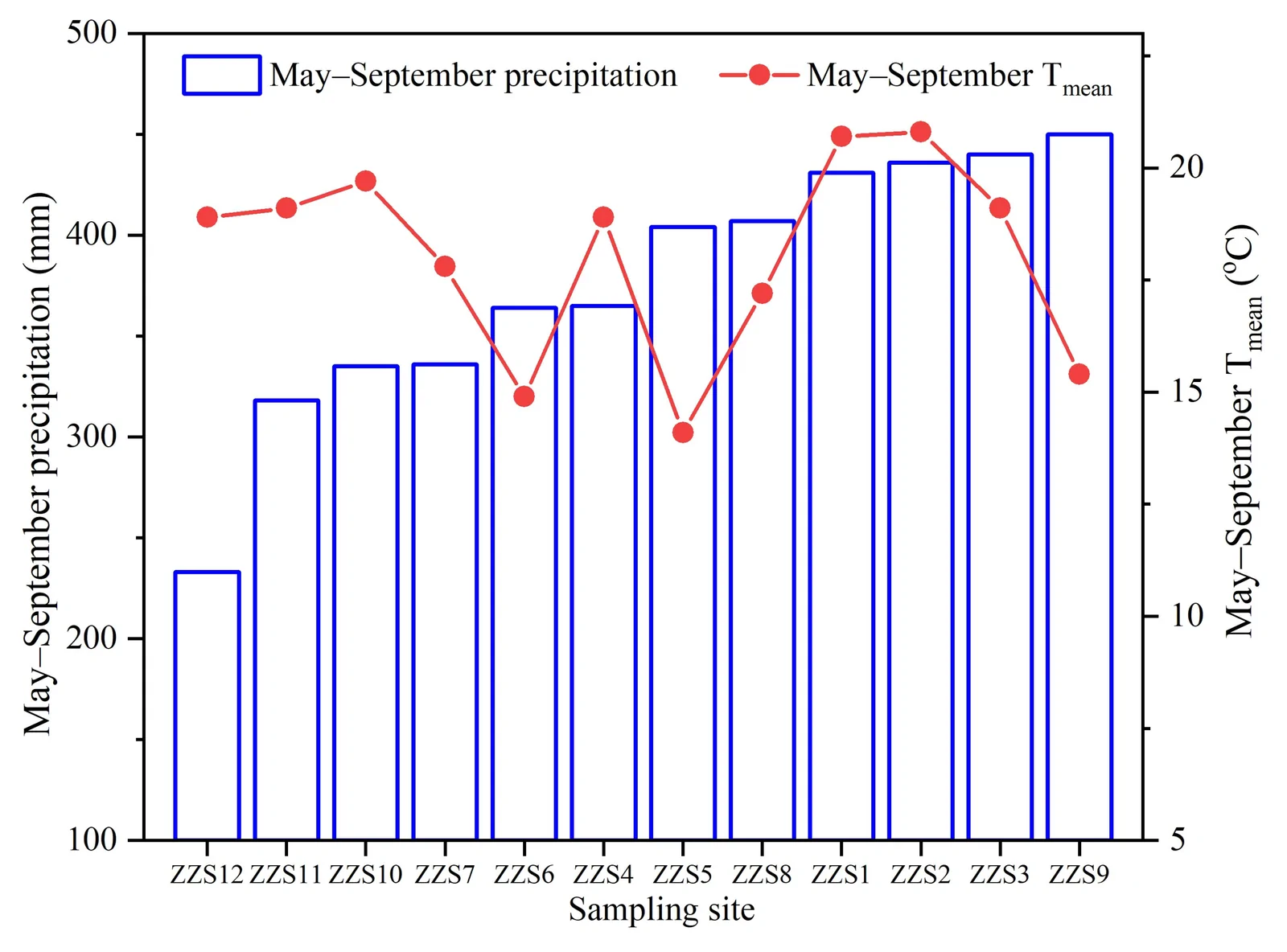
May–September (growing season) total precipitation and mean temperature (T_mean_) along a precipitation gradient across the sampling sites of the Mongolian pine plantations. Climatic values for ZZS7, ZZS10 and ZZS11 represent those for the sites ZZS7-1 to ZZS7-3, ZZS10-1 to ZZS10-3 and ZZS11-1 to ZZS11-2, respectively, because the sites in each cluster are located close to one another and thus are assumed to share the same climatic conditions.

The stem growth (reflected by RW) of Mongolian pine trees did not appear to be constrained by precipitation alone at the broad regional scale, although most sampling sites are water-limited, with growing-season precipitation below 450 mm (Figure 9). In other words, the spatial distribution of precipitation in our study area did not obviously affect the spatial pattern of Mongolian pine stem growth. This finding is not consistent with previous reports that tree growth has decreased gradually along a precipitation gradient from high to low [50,51]. The lower impact of precipitation on stem growth observed in our study may be due to the root characteristics of trees. Mongolian pine trees have relatively long roots that can extend more than 1 m underground [52]. Therefore, trees may access deep soil water to mitigate water stress, which could weaken the water limitation on growth at sites where precipitation is relatively low, thus potentially explaining the reduced growth differences among sites with contrasting precipitation. Although the role of precipitation in tree growth is limited, we found that drought intensity (represented by DMI, which integrates precipitation and temperature) exerted significant adverse impacts on the stem growth of Mongolian pine at a broad spatial scale. The significant negative correlation between RW and growing season temperature implied that high temperature can elevate water stress through increasing transpiration and thus reduce tree growth. This is consistent with previous studies reporting exacerbated water stress and reduced growth under warmer conditions in other water-deficit regions [20,53,54]. The positive relationship between stem growth and DMI across sites revealed by our study suggests that Mongolian pine plantations in drier sites in northern China might have experienced water stress-induced growth decline, and this trend will be further aggravated under climate warming.

In contrast to the stem growth of Mongolian pine trees, their canopy activity (represented by remotely sensed NDVI) responded strongly to growing season precipitation on a broad scale in our study area, which agrees well with previous studies documenting strong precipitation limitation on vegetation productivity (reflected by remote sensing indicators) in semi-arid and arid regions [27,55,56]. Canopy activity appears to be sensitive to shallow soil water, which is likely replenished primarily by precipitation, especially in water-deficit areas [57]. Therefore, precipitation conditions at a site, typically represented by multiple-year mean precipitation, may exert a strong influence on canopy activity, and a strong association between precipitation and canopy activity would likely emerge at large spatial scales encompassing many sites with contrasting precipitation regimes. Unlike canopy activity, stem growth may also be influenced strongly by deep soil water and is thus less dependent on shallow soil water, which is replenished mainly by precipitation [58,59]. Because of the differences in water response between canopy activity and stem growth, the former metric followed the precipitation gradient well from low to high, but the latter one did not (Figure 1 and Figure 9), which could obviously attenuate the relationship between these two growth-related metrics of Mongolian pine plantations across a broad region in northern China.

One caveat of using mean tree-ring width (RW) to compare sites is that the eight-year windows may represent different calendar periods at different sites, owing to differences in their establishment years. Averaging over eight years largely dampens interannual climate signals, but decadal-scale climatic differences cannot be fully ruled out. For such a bias to account for the observed patterns of among-site differences, it would need to operate systematically and in the same direction across all sites, whereas climatic variation among establishment periods is expected to affect sites idiosyncratically. Furthermore, strong correlations (*r* = 0.815, *p* < 0.001; Figure S1) between RW and mean basal area increment (BAI), calculated from tree-ring width measurements over the available measurement periods across the sites, indicated the robustness of the RW results. Therefore, we consider the calendar-year differences in RW calculation among sites unlikely to alter the main conclusions.

### 3.2. Differential Climate Sensitivity Weakens Stem–Canopy Coupling at the Site Scale

At the individual-site scale, the positive correlations between RWI and both growing season precipitation and drought index (scPDSI) at most sites underscore the critical role of water availability in the stem growth of Mongolian pine plantations, consistent with previous reports that water deficit strongly constrains tree growth in semi-arid and arid areas [45,60]. Insufficient water may negatively affect stem growth through weakening photosynthetic carbon assimilation caused by stomatal closure and impeding cambium activity induced by turgor loss [61,62]. Such adverse impacts on Mongolian pine trees could be particularly strong under more severe water stress, as indicated by more significant correlations of RWI with growing-season precipitation and scPDSI in drier sites. Furthermore, RWI correlated more strongly with scPDSI (incorporating precipitation and temperature) than with precipitation alone at most sites, indicating that temperature exacerbated the effect of water stress on tree growth at the individual-site scale, consistent with the broad-scale pattern. The temperature-induced increases in water stress limiting stem growth at both contrasting scales, revealed by the current study, suggest that the projected temperature rise would exert increasingly adverse effects on Mongolian pine plantations in northern China, particularly at those sites with severe water deficits.

Compared with the relationships between stem growth and climate, weaker interannual correlations of NDVI_g_ with both growing season precipitation and scPDSI were found at the individual-site scale, indicating that short-term water variability might have limited roles in modulating the canopy activity of Mongolian pine plantations. The negligible roles could be further demonstrated by relatively high resistances in coping with extreme droughts for NDVI_g_ at most sites. The weak impacts of climate on canopy activity at short time scales were also reported in several other studies [63,64]. Canopy activity was quite sensitive to water variability and could quickly recover through precipitation replenishment after drought and thus commonly suffered less adverse impacts from water stress than stem growth, which was deeply influenced by water deficit due to impaired cambium activity [65,66]. Furthermore, trees may allocate more resources to canopy leaves to maintain photosynthesis and other metabolism activities during drought, thus potentially reducing the resources available for stem growth [16,63]. This resource allocation strategy under extreme conditions may further alleviate the negative impacts on tree canopy [42]. Therefore, the greater vulnerability of stem growth than canopy activity to climate variability at the individual-site scale could lead to pronounced differences in growth variability (as reflected by the coefficient of variation in the growth indicators) between the two components and ultimately decouple their relationships.

In addition to differences in climate responses during the current growing season, inconsistencies in response to pre-growing-season climate may also partially contribute to the decoupling between stem growth and canopy activity. Stem growth (RWI) exhibited significant (*p* < 0.05) positive correlations with pre-growing-season (previous October to current April) mean scPDSI (sites ZZS10-1~ZZS10-3 and ZZS11-1~ZZS11-2) and precipitation (sites ZZS10-2~ZZS10-3) at several dry sites, but canopy activity (NDVI) lacked such responses (Figure 5 and Figure 6). The lagged climate responses of stem growth have also been reported in other water-deficit environments, likely attributable to the promoting effects of pre-growing-season moisture conditions on cambial reactivation and xylem differentiation in spring by maintaining adequate turgor pressure, thereby benefiting subsequent growth [67,68]. Although the divergent responses to pre-growing-season climate conditions between stem growth and canopy activity were detected at only a few dry sites, the legacy effects of climate on stem growth may play an important role in modulating its relationship with canopy activity and influencing the carbon dynamics of Mongolian pine plantations in water-limited areas.

It is worth noting that the use of NDVI as a proxy for canopy activity may introduce uncertainty at sites with young, low-density plantations, although this remotely sensed vegetation index has been widely used for monitoring canopy activity [69,70,71]. In low-density forest stands, NDVI-derived canopy activity signals of forests can be contaminated by contributions from bare soil and understory vegetation [72]. However, the contamination from soil background should have minimal impact on the interannual variations in NDVI signals in this study, as demonstrated by comparing them with those of the soil-adjusted vegetation index (SAVI), which has been suggested to effectively minimize the impacts from the soil background [72]. Even at site ZZS6, which had the lowest forest stand density among the sites analyzed and a mean tree age of only 12 years (Table 1), a high interannual correlation was observed between NDVI and SAVI (*r* = 0.968, *p* < 0.001; Figure S2), confirming that soil background effects have minimal influence on the interannual variability of NDVI, even in the young, low-density plantations. Nevertheless, because SAVI corrects for soil but not understory contributions, we acknowledge that residual understory contributions cannot be entirely excluded, although understory cover at the Mongolian pine plantation sites is relatively sparse based on our field surveys. Future studies based on higher-resolution imagery would help further separate canopy from understory signals.

A further caveat is the limited temporal sample size for analyzing the interannual correlations of NDVI with RWI and climate variables, with a time span of only 8 years (2016–2023), which was constrained by the availability of the remote sensing records and their overlap with tree-ring data. This limitation may reduce the statistical power and increase the uncertainty of the correlation results. Nevertheless, relatively short overlapping periods are common in examining the relationships between tree-ring and remotely sensed metrics, owing to the limited archives of high-resolution remote sensing data [16]. Longer time series of these variables would therefore enable more rigorous examination of their relationships in future studies.

Moreover, the observed RWI–climate response patterns in this study may be confounded by ontogenetic and genetic factors, as the investigated plantation-grown trees are relatively young, many younger than 40 years. Given that biological factors could strongly affect tree growth and its relationship with climate in juvenile trees, the use of a detrended tree-ring parameter (i.e., RWI) in the growth–climate correlation analysis may partially reduce such confounding effects, as detrending removes age-related biological trends in raw tree-ring series [73]. However, ontogenetic and genetic influences cannot be fully eliminated through the detrending procedure, and thus some caution is needed when interpreting RWI–climate relationships.

### 3.3. Implications for Forest Carbon Sinks

This study revealed that stem growth was not significantly (*p* > 0.05) correlated with the NDVI-based proxy for canopy activity in Mongolian pine plantations in northern China at both the broad spatial scale and the individual-site scale, indicating substantial inconsistencies between stem and canopy dynamics. Accurate projections of long-term forest growth trends, which are closely associated with forest carbon sequestration, thus require the simultaneous examination of multiple tree components by combining dendroecological and remote sensing approaches. In recent years, canopy greenness enhancement, typically captured by remotely sensed vegetation indices, has been frequently reported at both regional and global scales [69,70,71]. Nevertheless, this does not necessarily mean an increase in forest carbon sink, since most studies lacked concurrent investigations of stem growth dynamics, which represent long-term carbon stocks in forest ecosystems. The inconsistencies between stem growth and canopy activity observed in our study underscore the importance of comprehensively assessing forest carbon sequestration dynamics and further revealing the underlying drivers. This study suggested that divergent climate responses could decouple stem growth from canopy-level growth, highlighting the need for a more thorough understanding of forest growth dynamics in response to climate change. Stem growth (represented by tree-ring indicators) responded strongly to temperature-induced water stress and extreme droughts, indicating that the long-term forest carbon sink will be increasingly constrained under climate warming and more frequent drought extremes, at least for the pine plantations in our study region. In contrast, canopy-level growth dynamics (reflected by NDVI_g_) were weakly correlated with climate variability and exhibited only weak fluctuations in response to climate extremes, in line with a recent report [63], suggesting that canopy dynamics might be less affected by short-term climate fluctuations. However, strong correlations between NDVI_g_ and average hydroclimatic conditions across the Mongolian pine plantations suggest that shifts in climate regimes driven by long-term climate change (e.g., warming and drying) could profoundly alter canopy-level growth dynamics and the associated carbon sequestration processes. Therefore, comprehensive investigations into the growth dynamics of multiple tree components and their responses to climate change are critical for accurately projecting forest carbon sinks.

## 4. Materials and Methods

### 4.1. Study Area

**Figure 10:**
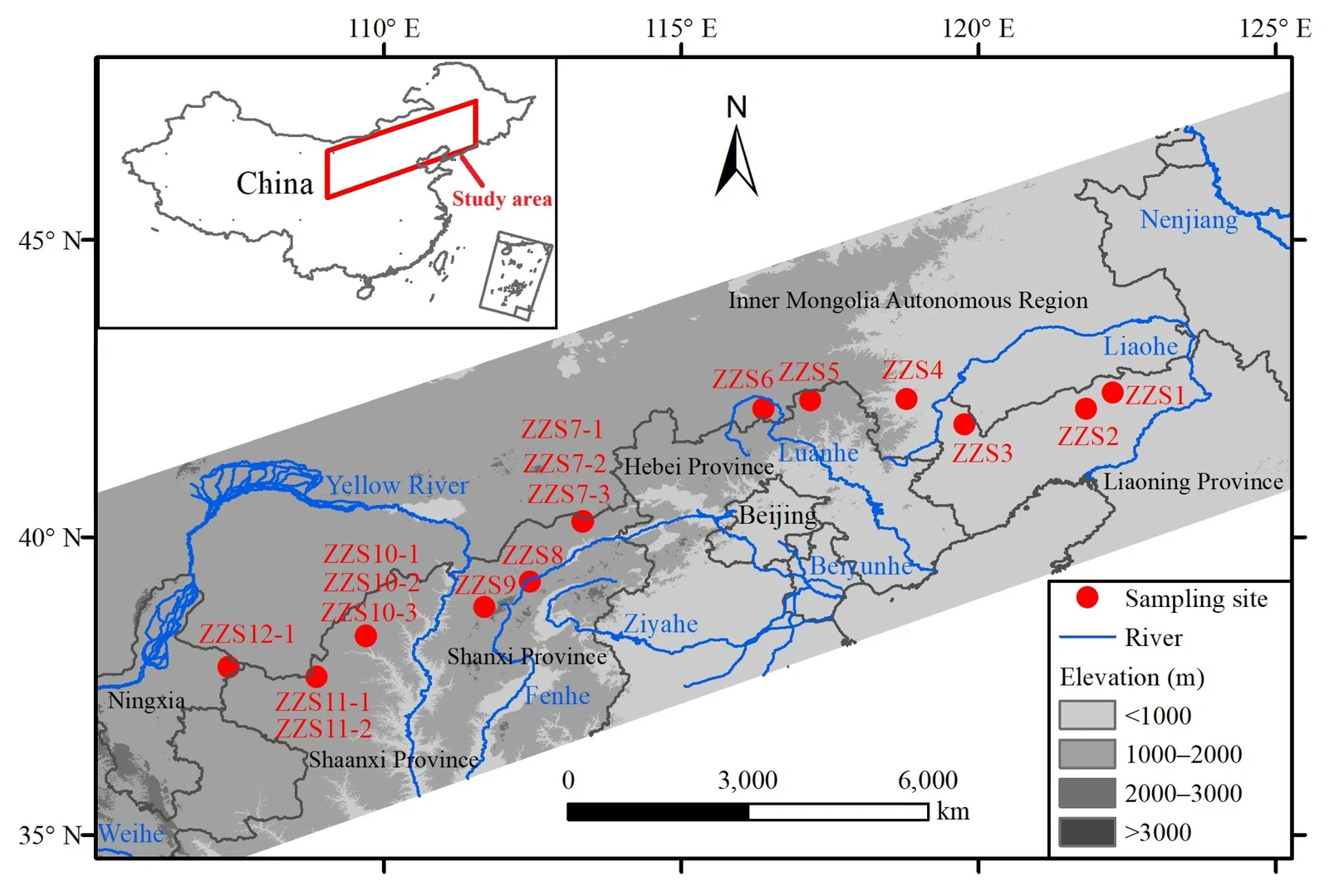
The spatial distribution of the 17 sampling sites of Mongolian pine plantations in northern China. The sites ZZS7-1 to ZZS7-3, ZZS10-1 to ZZS10-3 and ZZS11-1 to ZZS11-2 appear as single dots on the map because the sites are located close to one another.

The study area is located in the northern part of China, covering a broad region that extends from the northeast and north to the northwest (Figure 10). The region is characterized by a temperate monsoon climate, with most of the annual total precipitation falling between May and September. The climate conditions vary notably among the sampling sites investigated, with May–September total precipitation ranging 233 to 450 mm and May–September mean temperature from 14.1 to 20.8 °C (Figure 9). The elevations of the sites are between 128 and 1484 m a.s.l. (Table 1).

Mongolian pine (*Pinus sylvestris* var. *mongolica* Litv.) is a variety of Scots pine (*Pinus sylvestris* L.). Because of its tolerance to drought, this species has been widely used for establishing plantations in water-limited regions of northern China over recent decades, and the plantations now extend across more than 300 counties from the northeastern to northwestern parts of the country [74]. At the study sites, Mongolian pine shows better growth at relatively humid sites, whereas some trees at dry sites (mainly in northwest and north China) suffer from canopy dieback and even mortality. In relatively humid environments, Mongolian pine plantations are adjacent to broadleaf species such as *Populus pseudo-simonii Kitag.* and *Populus alba* × *Populus berolinensis* or to farmland. In contrast, plantations in dry environments are surrounded by sparse grassland or sandy land.

### 4.2. Acquisition of Tree-Ring Width Data

#### 4.2.1. Tree-Ring Measurement

During mid to late October 2023, we selected 17 sites of Mongolian pine plantations for tree-ring sampling across the desertification control zone in northern China, from the northeast to the northwest (Figure 10). For each site, we established a quadrat with a size of 25 m × 25 m in the Mongolian pine plantations. In the quadrat, tree-ring cores of 13 to 26 trees, with one or two cores for each tree, were obtained at breast height (about 1.3 m above the ground) using an increment borer (Table 1). Altogether, 614 tree-ring cores from 311 trees were sampled for the 17 sites. In addition, the diameters at breast height of the trees in each quadrat were measured, and the stand density was calculated.

The obtained tree-ring cores were then taken to the laboratory for further analysis. After being dried in shady and cool conditions, the tree-ring cores were fixed in a wooden trough and polished using sandpapers of 600 and 1200 mesh to make the ring boundaries on the cores clearly visible. Using the software WinDENDRO, Regular 2019 version (Regent Instruments Inc., Quebec City, QC, Canada), tree-ring widths of the tree-ring cores were measured and then cross-dated by comparing and matching the extremely low and high values of ring-width series curves. The measured and cross-dated results of the tree-ring widths were then checked using the COFECHA program (version 6.06P) [75]. Because tree-ring sampling occurred during mid- and late October 2023, when the ring formation for the year had nearly finished, the 2023 data were included for analysis.

#### 4.2.2. Chronology Construction

The dated tree-ring width measurements were fitted using a cubic spline with a 50% frequency-response cutoff equal to two-thirds of the series length and were performed by the ARSTAN program (version 41d) [73]. The detrended tree-ring width indices of each core were then obtained through an approach whereby the actual tree-ring width measurements were divided by the corresponding fitted value, with an aim of removing or alleviating the variances related to biological trends and preserving the climate-related information. All the detrended tree-ring width series within each site were averaged to develop site tree-ring width chronologies using the bi-weight robust means method [25]. Several statistics were used to assess the resulting chronologies, including mean correlation between tree-ring cores (*R*), mean sensitivity (MS), signal-to-noise ratio (SNR), expressed population signal (EPS), and first-order autocorrelation (AR(1)) [25,73]. The reliable period of each site chronology was identified as the interval at which the EPS exceeded 0.85 [76]. The tree-ring width indices (RWIs) during the reliable periods were used to examine the interannual correlations between stem growth and climate at the individual-site scale. To compare the mean stem growth among the sites while controlling for potential age effects, the tree-ring widths of the first eight years of the cores (only series containing complete data for these years were used) were averaged for each site (RW). The first eight years were selected for analysis to match the age of the youngest Mongolian pine plantation (ZZS12; Table 1), which enables a direct comparison of the mean stem growth among all the sites during the same cambial-age period. Using the first eight years of tree-ring widths can also reduce the confounding effects of competition among trees within a site, as these young ages have not yet reached canopy closure, and thus this approach better reflects intrinsic site productivity than the use of tree-ring widths at older ages. In addition, to test the robustness of the RW results, we calculated basal area increment (BAI) at each site using tree-ring width measurements over the available measurement periods [12].

### 4.3. Acquisition of Remotely Sensed Normalized Difference Vegetation Index Data

The Sentinel-2A/B data were used to reflect canopy activity and growth behaviors. The Sentinel-2A and 2B satellites were respectively launched in June 2015 and March 2017, by the European Space Agency [77,78]. Each satellite provides multispectral data covering the globe with a 10-day revisit time; thus, the data are available every 5 days after the launch of the Sentinel-2B satellite. Each image contains 13 spectral bands, with spatial resolutions between 10 and 60 m. To align with the period of tree-ring data, the image data of the two satellites between May and September (representing the vegetation growing season) from 2016 to 2023 were obtained from the Google Earth Engine (GEE) platform. Given the temporal data available on the platform, Sentinel-2 Level-1C data were used for the period 2016–2018, and Level-2A data for the period 2019–2023. The spectral reflectance values of the red and near-infrared bands for each image with a spatial resolution of 10 m were used to calculate the normalized difference vegetation index (NDVI) through the GEE platform. The formula for calculating NDVI is as follows [79]:

(1) $$\mathrm{NDVI}=\frac{\mathrm{NIR}-R}{\mathrm{NIR}+R}$$

where NIR and R denote the spectral reflectance values in the near-infrared and red bands, respectively.

To assess potential inconsistencies between the NDVI data derived from Sentinel-2 Level-1C (2016–2018) and Level-2A (2019–2023) images, we used the Pettitt test [80] to detect possible breakpoints at the 2018–2019 transition for each site. The results were all non-significant (*p* > 0.05), indicating that the change in data source did not introduce a statistically detectable discontinuity.

To examine the canopy activity in the tree-ring sampling sites, the spatial extents of the Mongolian pine plantations were determined by identifying their image features and delineating their boundaries on Google Earth high-resolution imagery based on field investigations. Then, the vectorized boundaries of the pine plantations were used to extract the NDVI data from Sentinel-2A/B images for each site. To alleviate the potential effects of cloud contamination, the monthly NDVI data were obtained by selecting the maximum value for each month. The NDVI data from May to September were then averaged to represent the growing season NDVI (NDVI_g_). In addition, the NDVI_g_ was further detrended using linear regression in order to better isolate climatic effects from successional trends, for the following reasons: (1) NDVI_g_ at all investigated sites exhibited significant (*p* < 0.05) linear trends during the period 2016–2023, which were most likely associated with stand development (i.e., canopy closure and biomass accumulation), as most sites are relatively young plantations; (2) the NDVIg trends were unlikely to be climatically driven, because growing-season precipitation and temperature showed no significant linear trends over the same period at any of the investigated sites, although other non-climatic factors (e.g., phenological drift) cannot be fully excluded; (3) given the short period of the NDVIg series (8 years, 2016–2023), more flexible detrending approaches (e.g., spline or higher-order polynomials) would result in a substantial loss of degrees of freedom and carry a high risk of overfitting; and (4) this detrending is conceptually analogous to the standardization of tree-ring width series to derive RWIs, with both aiming to remove low-frequency, non-climatic signals, and such parallel treatments of vegetation-index and tree-ring data have been commonly applied in previous studies [81,82]. Overall, linear detrending removes the presumably successional trend from NDVI_g_ and thus enables a more robust evaluation of its interannual correlations with RWI and with climate variability. To assess the representativeness of the NDVI extracted from the vectorized boundaries, we also calculated the mean NDVI of a 3 × 3 pixel window centered on each tree-ring sampling site and found that the correlation between the NDVI values derived from these two approaches was strong (Figure S3).

Considering that the NDVI signals of some young, low-density plantation stands may be contaminated by soil background, we also calculated the soil-adjusted vegetation index (SAVI) for the site with the lowest stand density (ZZS6; Table 1) to assess the magnitude of these effects. SAVI was developed by Huete (1988) [72] to minimize soil background effects on vegetation indices, and it is calculated as follows:

(2) SAVI=[(NIR−R)/(NIR+R+L)]×(1+L)

where *L* is a soil adjustment factor, which is typically set to 0.5 as recommended by Huete (1988) [72].

### 4.4. Acquisition of Climate Data

The monthly climate data, including mean temperature (T_mean_), total precipitation and mean self-calibrating Palmer Drought Severity Index (scPDSI), were derived from the gridded CRU TS v4.08 dataset with a spatial resolution of 0.5° × 0.5° [83]. The data from the grid cells near each tree-ring sampling site were employed. The monthly climate data were then aggregated over the current May–September period (i.e., growing season) and from the previous October to the current April (i.e., pre-growing season), and the resulting seasonal series were correlated with both RWI and NDVI at the individual-site scale. The scPDSI is a drought index based on a simplified soil water balance model driven by precipitation, temperature and potential evapotranspiration and has been successfully used for monitoring changes in drought conditions over time in many regions [84]. Lower scPDSI values indicate more severe drought. However, because scPDSI expresses anomalies relative to the local climatological baseline, it does not directly reflect the long-term aridity background at a given site. To compare the multi-year mean water stress conditions among the sites investigated, we employed the De Martonne drought index (DMI) developed by De Martonne (1926) [85]. The DMI is calculated based on precipitation and temperature data and is suitable for characterizing long-term aridity conditions, but it is not well-suited to tracking aridity changes over time. The formula for obtaining DMI is as follows [85]:

(3) $$\mathrm{DMI}=\frac{P}{T+10}$$

where *P* and *T* denote total precipitation and mean temperature during the growing season (from May to September), respectively.

### 4.5. Calculation of Drought Resistance and Recovery

To assess the responses of the RWI and NDVI_g_ to extreme droughts, we calculated drought resistance and recovery following the method developed by Lloret et al. (2011) [12]. Since the common period (2016–2023) for RWI and NDVI_g_ was relatively short, the year with the lowest scPDSI for each site was determined as the extreme drought year. In calculating drought resistance (*Rt*) and recovery (*Rc*), the RWI/NDVI_g_ data two years before and after extreme droughts were used based on previous studies that reported a drought legacy effect in forest ecosystems persisting for 1 to 2 years [86]. The formulas for calculating the two indicators are as follows [12]:

(4) $$Rt=\frac{Dr}{\bar{Dr_{\mathrm{pre}}}}$$

where *Dr* and $\bar{Dr_{\mathrm{pre}}}$ denote RWI (or detrended NDVI_g_) in the extreme drought year and the average in the two years before the drought event, respectively.

(5) $$Rc=\frac{\bar{Dr_{\mathrm{post}}}}{Dr}$$

where $\bar{Dr_{\mathrm{post}}}$ denotes the average of RWI (or detrended NDVI_g_) in the two years after the drought event.

### 4.6. Statistical Analysis

Pearson correlation analysis was used to examine the relationships between stem growth indicators (RW and RWI) and canopy activity indicators (NDVI_g_ and detrended NDVI_g_), as well as their respective correlations with climate variables. A one-way analysis of variance (ANOVA) was used to determine the differences in mean resistance and recovery between stems and canopies.

## 5. Conclusions

No statistically significant (*p* > 0.05) relationships were found between stem growth (reflected by tree-ring indicators) and canopy activity (remotely sensed NDVI_g_) at both broad regional and individual-site scales for Mongolian pine plantations in northern China. The weak associations might be due to the divergent climate responses of these two key components of trees. At the broad spatial scale, stem growth was severely affected by temperature-induced water stress, whereas canopy activity responded strongly to growing season precipitation. At the specific site level, stem growth exhibited greater vulnerability to short-term hydroclimatic variability and extreme droughts during the growing season than canopy activity, and such adverse effects for stem growth were especially pronounced at drier sites. In addition, stem growth was more affected by pre-growing-season hydroclimatic variability relative to canopy activity at several dry sites. This study clarified the underlying causes of decoupled relationships between stem and canopy dynamics for the Mongolian pine trees from the perspective of climate drivers. The uncertainties in linkages between stem and canopy dynamics may also arise from other factors, such as the different timings of xylem formation and canopy development and the mismatch in spatiotemporal resolutions between the tree-ring and remote sensing data reflecting these two types of processes; future studies are required to clarify these issues. Further research incorporating physiological data (such as leaf stomatal conductance and xylem hydraulic traits) is also needed to reveal the underlying mechanisms of the divergent relationships between these two processes in trees under climate change.

## Figures and Tables

**Table 1:** Information on the tree-ring sampling sites of the Mongolian pine plantations in northern China.

Sampling Site	Latitude (°N)	Longitude (°E)	Elevation (m)	Stand Density (Number/25 m × 25 m)	DBH (cm)	Tree-Ring Cores/Trees	Mean Tree Age (Year)	Mean Ring-Width (mm)
ZZS12	37.820	107.404	1356	100	8.36 ± 0.22	36/18	8	2.89
ZZS11-1	37.649	108.875	1302	126	10.73 ± 0.37	42/21	34	1.36
ZZS11-2	37.649	108.880	1288	65	17.31 ± 1.23	32/17	25	2.17
ZZS10-1	38.339	109.707	1067	75	21.26 ± 0.88	36/18	29	3.23
ZZS10-2	38.337	109.709	1067	75	18.77 ± 1.05	26/13	30	2.27
ZZS10-3	38.335	109.709	1070	100	18.23 ± 0.79	33/17	28	2.49
ZZS7-1	40.252	113.347	1139	155	10.86 ± 0.31	26/13	21	2.07
ZZS7-2	40.263	113.352	1173	95	12.79 ± 0.34	36/18	23	2.14
ZZS7-3	40.265	113.359	1149	91	13.76 ± 0.43	38/19	21	2.35
ZZS6	42.157	116.394	1274	40	16.44 ± 0.43	39/20	12	4.87
ZZS4	42.326	118.802	684	83	15.39 ± 0.56	51/26	38	1.63
ZZS5	42.300	117.181	1458	84	19.56 ± 0.73	34/17	30	2.76
ZZS8	39.250	112.461	1090	172	14.07 ± 0.41	36/18	29	1.93
ZZS1	42.432	122.269	128	66	22.27 ± 0.70	33/18	35	2.13
ZZS2	42.160	121.814	286	74	17.39 ± 0.52	32/16	37	1.47
ZZS3	41.893	119.777	752	71	16.84 ± 0.63	50/25	33	2.12
ZZS9	38.826	111.698	1484	85	13.85 ± 0.47	34/17	14	3.68

Values preceding and following ± represent the mean and 1 SD, respectively.

**Table 2:** Statistical characteristics of the tree-ring width chronologies from the sampling sites of the Mongolian pine plantations in northern China.

Sampling Site	Span Time	*R*	MS	SNR	EPS	AR(1)
ZZS12	2013–2023	0.61	0.28	43.67	0.98	−0.38
ZZS11-1	1982–2023	0.66	0.39	54.08	0.98	0.36
ZZS11-2	1990–2023	0.74	0.50	57.42	0.98	−0.04
ZZS10-1	1989–2023	0.79	0.34	97.28	0.99	0.12
ZZS10-2	1988–2023	0.73	0.42	53.97	0.98	−0.11
ZZS10-3	1989–2023	0.75	0.42	74.35	0.99	−0.03
ZZS7-1	1998–2023	0.30	0.16	7.94	0.89	−0.14
ZZS7-2	1996–2023	0.67	0.36	65.79	0.99	−0.23
ZZS7-3	1997–2023	0.58	0.20	41.31	0.98	−0.26
ZZS6	2010–2023	0.83	0.30	155.30	0.99	−0.41
ZZS4	1979–2023	0.49	0.30	40.53	0.98	0.42
ZZS5	1991–2023	0.74	0.24	83.37	0.99	0.24
ZZS8	1991–2023	0.46	0.18	24.32	0.96	0.22
ZZS1	1982–2023	0.48	0.26	21.55	0.96	0.52
ZZS2	1974–2023	0.40	0.27	14.53	0.94	0.16
ZZS3	1984–2023	0.61	0.41	51.07	0.98	−0.16
ZZS9	2007–2023	0.56	0.15	29.06	0.97	−0.21

*R* is mean correlation between tree-ring cores; MS is mean sensitivity; SNR is signal-to-noise ratio; EPS is expressed population signal; AR(1) is first-order autocorrelation.

## Data Availability

Data are available from the corresponding authors on reasonable request.

## Supplementary Materials

The following supporting information can be downloaded at: https://www.mdpi.com/article/10.3390/plants15213403/s1. Figure S1: Correlations of mean basal area increment (BAI) with RW (a) and growing-season NDVI_g_ (b) across the sampling sites of the Mongolian pine plantations. RW represents the mean tree-ring width over the first eight years of growth; Figure S2: Correlation between growing-season (May–September) normalized difference vegetation index (NDVI_g_) and soil-adjusted vegetation index (SAVI_g_) at ZZS6, the site with the lowest stand density; Figure S3: Correlations of growing-season (May–September) normalized difference vegetation index (NDVI_g_) between the forested area investigated and the 3 × 3-pixel area centered on each tree-ring sampling site.
