# Supplementary material for: Divergent Climate Responses Decouple Stem Growth from Canopy Activity Across Mongolian Pine Plantations in Northern China
Source: Plants (Basel). 2026 Nov 2;15(21):3403. doi: 10.3390/plants15213403 (PMC13765567; doi:10.3390/plants15213403)
Supplement: Supplementary file 1 [file plants-15-03403-s001.zip › plants-4573015-supplementary.pdf]

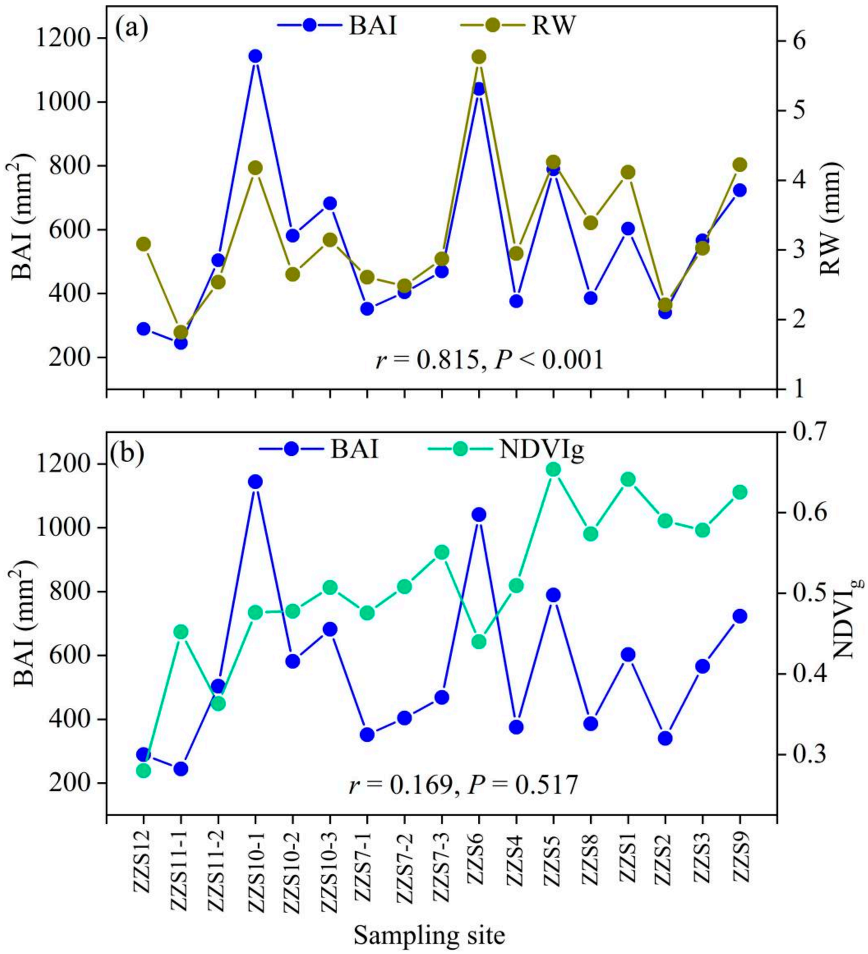

Figure S1. Correlations of mean basal area increment (BAI) with RW (a) and growing-season $NDVI_g$ (b) across the sampling sites of the Mongolian pine plantations. RW represents the mean tree-ring width over the first eight years of growth.

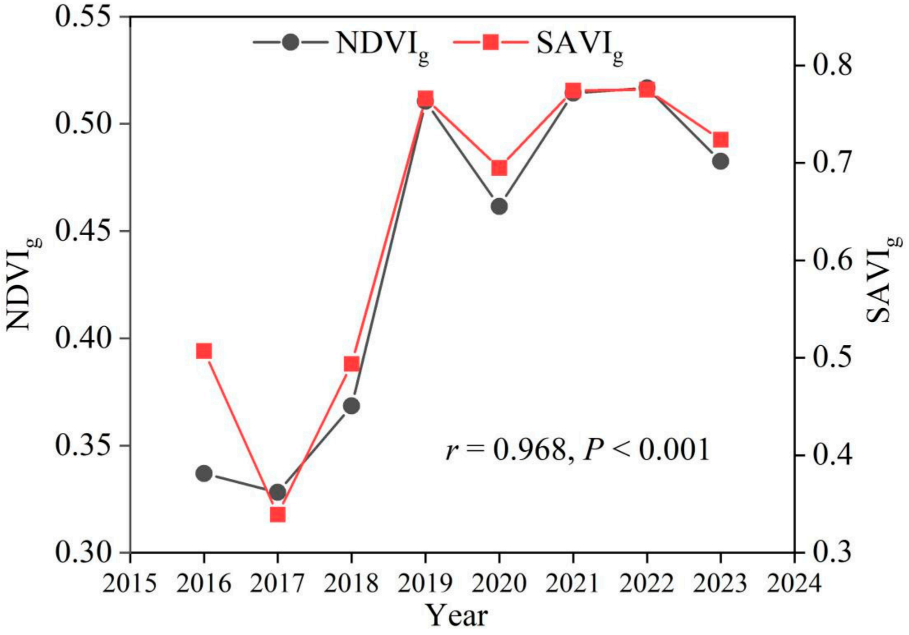

Figure S2. Correlation between growing-season (May–September) normalized difference vegetation index ($NDVI_g$) and soil-adjusted vegetation index ($SAVI_g$) at ZZS6, the site with the lowest stand density.

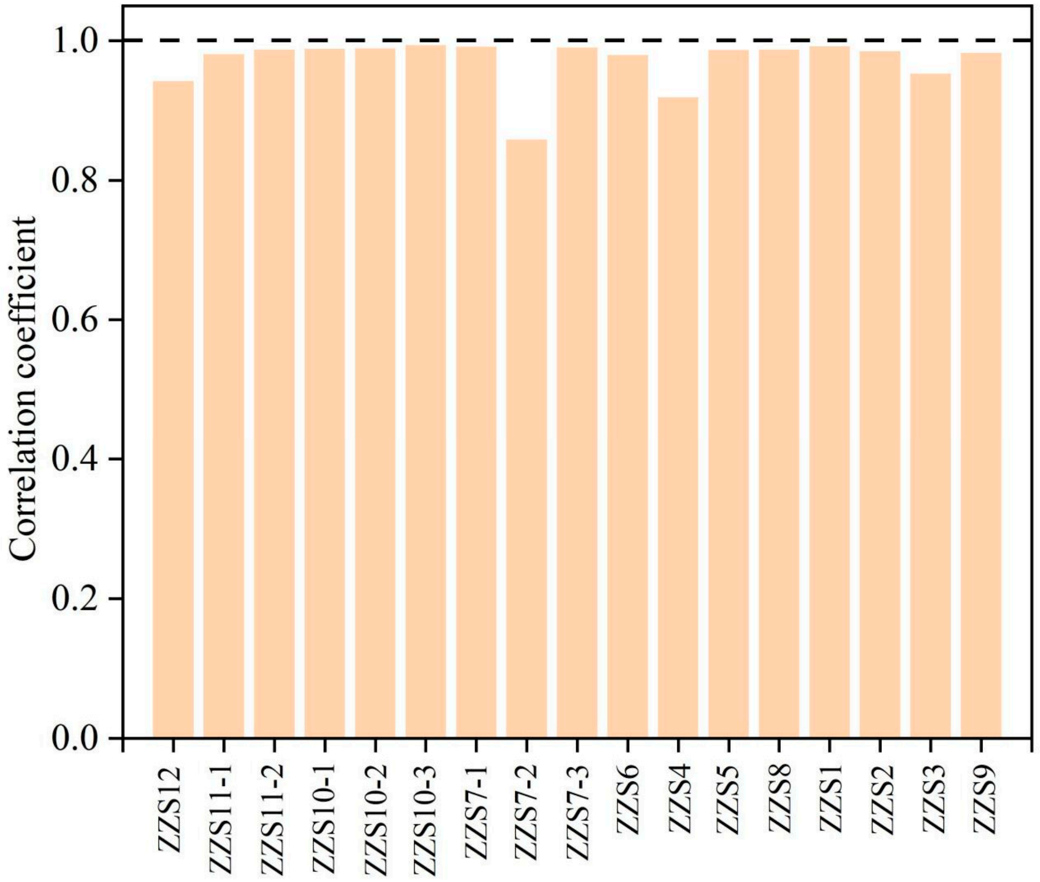

Figure S3. Correlations of growing-season (May–September) normalized difference vegetation index ($NDVI_g$) between the forested area investigated and the 3 × 3-pixel area centered on each tree-ring sampling site.
